# Tuberculous pneumonia-induced severe ARDS complicated with DIC in a female child: a case of successful treatment

**DOI:** 10.1186/s12879-018-3215-5

**Published:** 2018-07-03

**Authors:** Dong Tien Ngo, Phuc Huu Phan, Shoji Kawachi, Noriko Nakajima, Naoyuki Hirata, Akira Ainai, Thuy Thi Bich Phung, Dien Minh Tran, Hai Thanh Le

**Affiliations:** 1 National Children’s Hospital, Hanoi, Vietnam; 2 National Center for Global Health and Medicine, Tokyo, Japan; 3 National Institute of Infectious Diseases, Tokyo, Japan; 4 Teikyo University, Tokyo, Japan; 5 Department of Anesthesiology, Sapporo Medical University School of Medicine, South-1 West-16, Chuo-ku, Sapporo, Hokkaido 060-8543 Japan

**Keywords:** Tuberculous pneumonia, Pediatric acute respiratory distress syndrome, Recombinant soluble human thrombomodulin

## Abstract

**Background:**

Tuberculous (TB) pneumonia can induce acute respiratory distress syndrome (ARDS). Although TB pneumonia is one of the causes of disease and death among children worldwide, the literature on TB pneumonia-induced ARDS is limited. We report herein on the successful treatment of a two-year-old female child with TB pneumonia-induced severe ARDS complicated with disseminated intravascular coagulation (DIC).

**Case presentation:**

A two-year-old Vietnamese female child with sustained fever and cough for 20 days was transferred to our hospital. She had severe dyspnea and a chest X-ray showed bilateral infiltration without findings of heart failure. After tracheal intubation, her oxygenation index (OI) and PaO_2_/FiO_2_ (PF) ratio were 29 and 60 mmHg, respectively. Mycobacterium tuberculosis was detected by real-time polymerase chain reaction (rPCR) assay of tracheal lavage fluid. She was diagnosed as having severe ARDS that developed from TB pneumonia. Anti-tuberculous therapy and cardiopulmonary support were started. However, her respiratory condition deteriorated despite treatment with high-frequency oscillating ventilation (HFO), vasopressor support, and 1 g/kg of immunoglobulin. On the third day after admission, her International Society on Thrombosis and Hemostasis DIC score had increased to 5. Recombinant human soluble thrombomodulin (rTM) was administered to treat the DIC. After the administration of rTM was completed, OI gradually decreased, after which the mechanical ventilation mode was changed from HFO to synchronized intermittent mandatory ventilation. The DIC score also gradually decreased. Plasma levels of soluble receptor for advanced glycan end products (sRAGE) and high mobility group box 1 (HMGB-1), which are reported to be associated with ARDS severity, also decreased. In addition, inflammatory biomarkers, including interferon-gamma (IFN-γ) and interleukin-6 (IL-6), decreased after the administration of rTM. Although severe ARDS (P/F ratio ≦ 100 mmHg) continued for 19 days, the patient’s OI and P/F ratio improved gradually, and she was extubated on the 27th day after admission. The severe ARDS with DIC was successfully treated, and she was discharged from hospital on day 33 post-admission.

**Conclusions:**

We successfully treated a female child suffering from TB pneumonia-induced severe ARDS complicated with DIC using multimodal interventions. (338/350).

## Background

Pulmonary tuberculosis (TB) is prevalent in developing countries, but is a rare cause of acute respiratory distress syndrome (ARDS) [1–3]. In pediatric populations, although ARDS is secondary to lung infections, including TB pneumonia [4], there have been few reports on severe ARDS complicated with disseminated intravascular coagulation (DIC) caused by TB pneumonia. We report herein the successful treatment of a female child suffering from TB pneumonia induced-severe ARDS complicated with DIC using multimodal interventions, including high-frequency oscillating ventilation, vasopressor support, anti-tuberculous therapy, intravenous immunoglobulin (IVIG) and recombinant soluble human thrombomodulin (rTM).

## Case presentation

A two-year-old Vietnamese female child had fever and cough 20 days before admission to our hospital. She visited a local hospital and was diagnosed with pneumonia. She was treated with meropenem and vancomycin for 15 days. Despite treatment, she developed increased fever (40 °C), persistent cough and general malaise. Her respiratory condition deteriorated and she was transferred to our hospital. Prior to hospitalization, she had been healthy and had developed normally. She had no immunodeficiency and no history of contact with anyone suffering from TB.

**Fig. 1:**
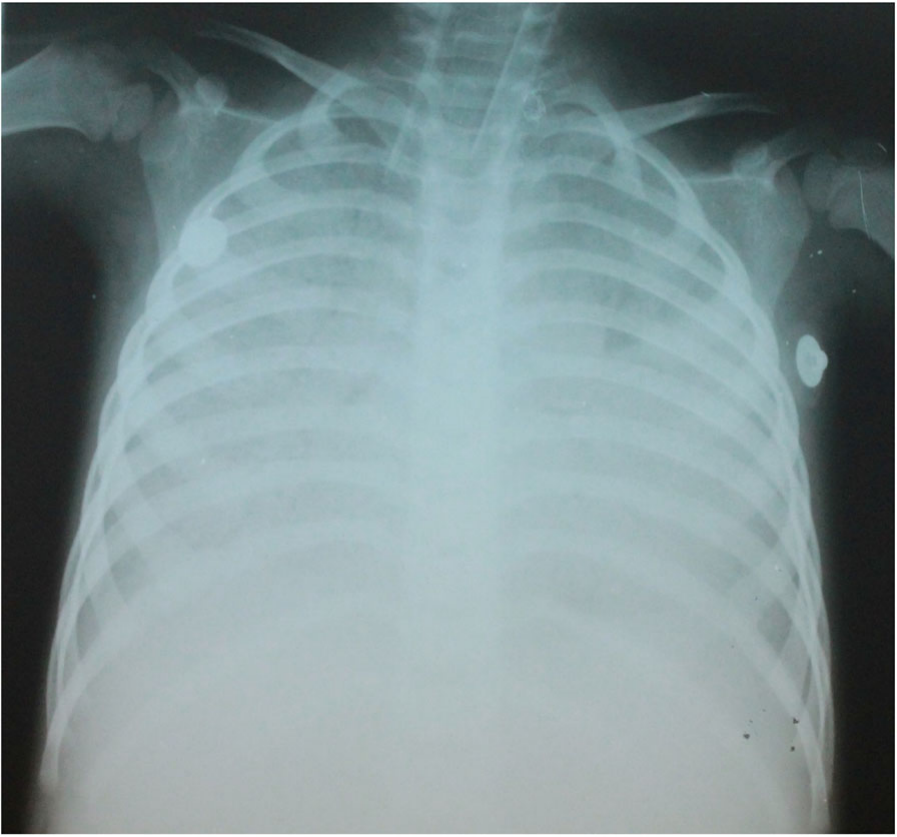
Chest X-ray on admission to PICU

**Fig. 2:**
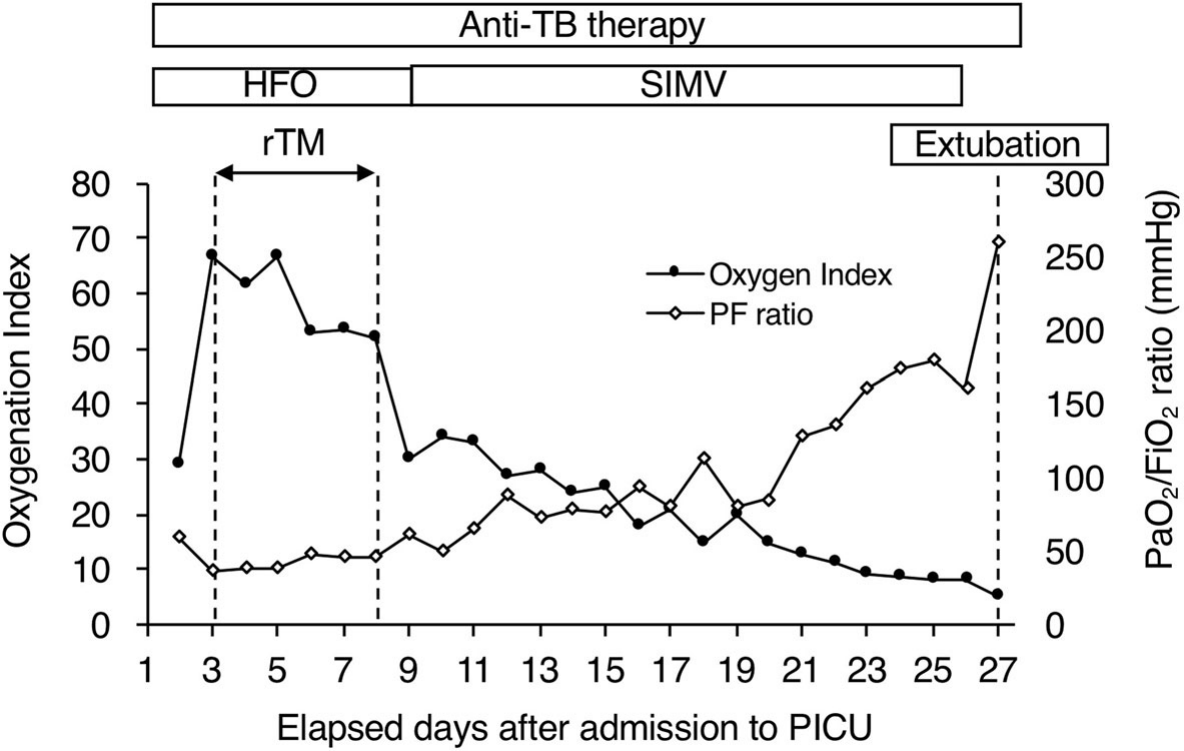
Changes in Oxygenation Index (OI) and PaO_2_/FiO2 (PF) ratio after admission to PICU

**Fig. 3:**
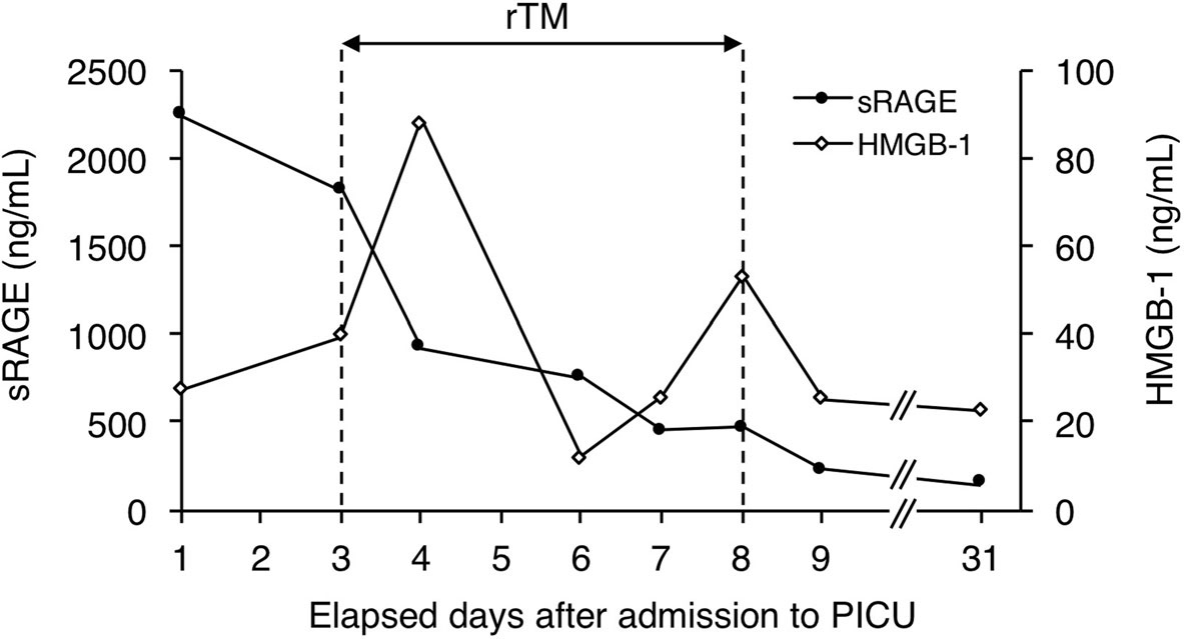
Changes in sRAGE and HMGB-1 before and after administration of rTM. sRAGE, soluble receptor for advanced glycan end products; HMGB-1, high mobility group box 1

**Fig. 4:**
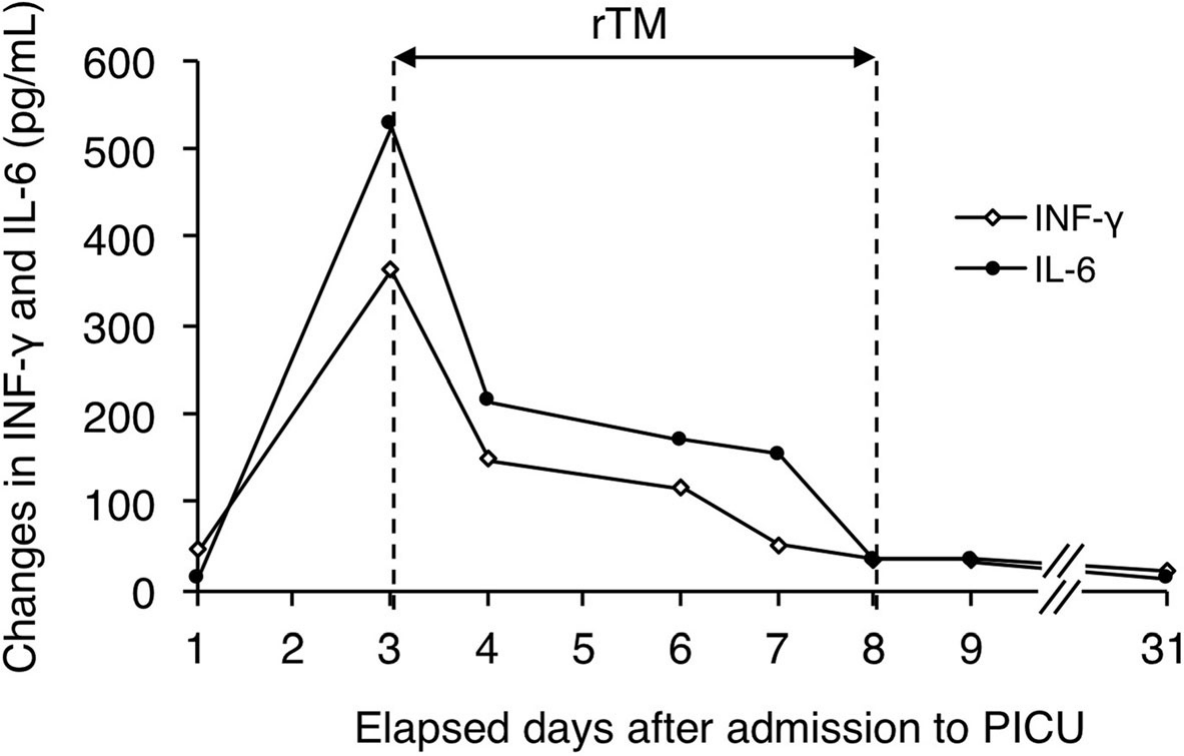
Changes in INF-γ and IL-6 before and after administration of rTM. INF-γ, interferon-gamma; IL-6, interleukin-6

**Fig. 5:**
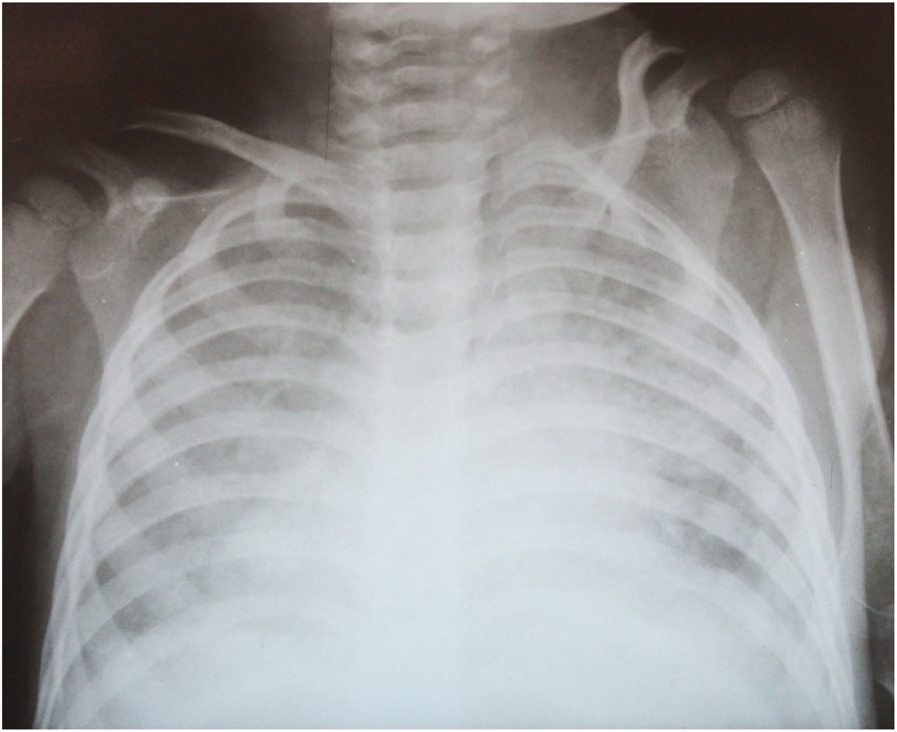
Chest X-ray just after extubation

Physical examination on admission showed a child with a weight of 10 kg, body temperature of 38.5 °C, blood pressure of 79/41 mmHg, and a heart rate of 157 beats/min. Her weight was reduced from 11 kg (25th percentile) to 10 kg (10th percentile) over the past month alone. Immediately after admission, tracheal intubation was performed because of severe dyspnea, and she was transferred to a pediatric intensive care unit (PICU). Arterial blood gas analysis in the PICU showed PaO_2_ of 60 mmHg, PaCO_2_ of 59 mmHg, and pH 7.27 under mechanical ventilation with FiO_2_ of 1.0, which resulted in a PaO_2_/FiO_2_ (P/F) ratio of 60 mmHg and an oxygenation index (OI) of 29 (Table 1). A chest X-ray showed bilateral infiltration without findings of heart failure (Fig. 1). Laboratory findings were as follows: white blood cell (WBC) count, 7000 cells/μl; red blood cell (RBC) count, 430 × 10^3^ cells/μl; platelet (PLT) count, 223 × 10^3^ cells/μl; aspartate aminotransferase (AST), 60 U/L; alanine aminotransferase (ALT), 13 U/L; blood urea nitrogen (BUN), 18.0 mg/dL; creatinine (Cre), 32 μmol/L; c-reactive protein (CRP), 7.3 mg/dL; fibrinogen, 2.79 g/L; and prothrombin time (PT), 16.1 s (Table 2). The results of a rapid influenza test were negative. Genomes of mycobacterium TB and cytomegalovirus (CMV) were detected in tracheal lavage fluid (TLF) by real-time polymerase chain reaction (rPCR) assay. Acid fast bacilli smear and culture were both positive. She was diagnosed as having severe ARDS that developed from TB pneumonia, and the standard anti-tuberculous drugs: rifampicin, 15 mg/kg/day; isoniazid, 10 mg/kg/day; pyrazinamide, 30 mg/kg/day; ethambutol, 20 mg/kg/day were administered. We also used colistin and levofloxacin for not only nosocomial infection, but also mycoplasma infection, which we could not rule out. However, her respiratory and circulatory conditions deteriorated despite high-frequency oscillating ventilation (HFO), vasopressor support (noradrenaline at 0.2 μg/kg/min), and administration of 1 g/kg of immunoglobulin. On the third day after admission, the patient’s International Society on Thrombosis and Hemostasis DIC score had increased to 5 (PLT, 153 × 10^3^ cells/μl; fibrinogen, 2.11 g/L; D-dimer, 5659 ng/mL; and PT, 23.3 s). For the treatment of DIC, 380 U/kg of rTM was administered as an intravenous drip infusion for 6 consecutive days. After administration of rTM was completed, OI gradually decreased, and the mechanical ventilation mode was changed from HFO to synchronized intermittent mandatory ventilation (Fig. 2). DIC score also gradually decreased. We measured plasma levels of soluble receptor for advanced glycan end products (sRAGE) and high mobility group box 1 (HMGB-1) before and after rTM administration, because increased levels of sRAGE and HMGB-1 are associated with death in patients with ARDS [5]. After administration of rTM, HMGB-1 and sRAGE decreased (Fig. 3), and inflammatory biomarkers, including interferon-gamma (IFN-γ) and interleukin-6 (IL-6), also decreased (Fig. 4). Throughout the administration of rTM, there was no bleeding-related and other adverse event. Although severe ARDS (P/F ratio ≦ 100 mmHg) was sustained for 19 days, the patient’s OI and P/F ratio improved gradually (Fig. 2), and she was extubated on the 27th day after admission. Although there was still a diffuse pattern in the chest X-ray just after extubation (Fig. 5), her fever went down and her respiratory condition was stable (P/F ratio: 325, OI: 3). Therefore, she was moved from the PICU to the infectious disease department. The severe ARDS with DIC was successfully treated, and she was discharged from hospital on the 33rd day after admission.

**Table 1:** Changes in vital sings

Days after admission	1^st^	3^rd^	4^th^	5^th^	6^th^	7^th^	8^th^	9^th^	10^th^	27^th^
Oxygenation Index	36	29	66	61.5	66.7	53	53.3	52	32	3
Ventilation mode	PRVC	HFO						SIMV		Extubation
Blood Pressure (mmHg)	79/41	82/51	94/49	89/45	109/60	100/49	103/66	105/60	75/45	98/60
Heart Rate (bpm)	126	157	149	127	128	128	158	165	156	130
Body Temperature (°C)	38.5	38.5	38.2	38.6	38.8	38.6	39	39.3	39.7	36.7

*PRVC* Pressure regulated volume control, *HFO* high frequency oscillating ventilation, *SIMV* synchronized intermittent mandatory ventilation

**Table 2:** Changes in laboratory data

Days after admission	1^st^	3^rd^	4^th^	5^th^	6^th^	7^th^	8^th^	9^th^	10^th^	27^th^
WBC (× 1000/μl)	7	6.7	3.7	6	5	6.6	10.5	12.2	7.7	13
Plt (×1000/μl)	223	153	76	65	60	67	81	142	123	291
AST (U/L)	60	70	92	92	87	87	73	73	95	170
ALT (U/L)	13	16	17	17	14	15	13	14	16	81
Cre (μmol/L)	32	50	45	52	47	43	41	48	41	39
BUN (mg/dL)	18	18	22.8	27	18.6	18	22	18	15	12
Fibrinogen (g/L)	2.79	2.11	2.05	2.1	2.3	1.53	0.8	0.75	2.22	4
D-dimer(ng/mL)	1140	5659	1212	1133	1316	1195	1250	6841	8442	916
PT (sec.)	16.1	23.3	20.9	19.4	16.6	18.4	22	16.4	13.7	10.9
DIC score	3	5	5	4	5	6	5	3	3	1
CRP (mg/dL)	7.3	13.0		12.2	15.8			4.7	3.9	2.6

## Discussion and conclusions

TB continues to be one of the causes of disease and death among children worldwide, particular in developing countries with a poor public health infrastructure [6, 7]. While TB-associated ARDS in adult patients has been reported [3], surveillance data for estimating the contribution of TB to pediatric ARDS remains limited [7]. Thus, it can be speculated that many pediatric patients with TB pneumonia induced-ARDS have not been reported in the literature. More robust data on the epidemiology of childhood TB are needed in order to determine not only the contribution to ARDS but also to develop pediatric-specific therapeutic strategies.

Previous reviews have indicated that pediatric TB is associated with a high prevalence of human immunodeficiency virus (HIV) infection [7, 8]. In the present case, the patient had no HIV infection or immunodeficiency. She also had no history of contact with anyone suffering from TB. Diagnosis of TB in children without a specific history is challenging because many childhood diseases can present with similar symptoms and signs, and bacteriological confirmation and specimen collection may be difficult [9]. Nevertheless, earlier diagnosis of and therapy for TB might have prevented proceeding to severe ARDS in the present case.

In this case, TB and CMV were detected in TLF on the PICU admission day by PCR. CMV also causes severe pneumonia [10]. In this case, 2.19 × 10^4^ /ml of CMV-DNA was detected in TLF on the day of admission. The copy number of CMV-DNA in TLF decreased to under the detection limit and CMV-DNA in blood was 2.16 × 10^3^ /ml without anti-CMV medication on the 8th day after admission. These levels of CMV-DNA copy numbers are observed also during CMV latent infection. So, we concluded that TB infection rather than CMV infection was the main pathogen of pneumonia that developed into severe ARDS in this case.

While a recent pediatric acute lung injury consensus conference [11] developed recommendations for therapeutic strategies for pediatric ARDS, the mortality rate in pediatric patients with ARDS has remained high, approximately 24% according to a recent meta-analysis [12]. In our case, immediately after diagnosis of TB pneumonia-induced ARDS, anti-TB therapy and interventions for ARDS, including lung protective support with HFO, administration of antibiotics and high-dose IVIG therapy to control inflammation, were started. However, the patient’s condition deteriorated and she developed DIC-related complications. As an anti-DIC therapy, rTM was administered. rTM is a new anticoagulant agent that is expected to have both anti-coagulopathy and anti-inflammatory effects [13]. In addition, the incidence of bleeding-related adverse events leading to discontinuation for rTM was significantly lower than that for heparin [13]. Previous clinical studies have demonstrated that rTM could improve not only mortality in patients with sepsis-induced DIC but also respiratory dysfunction in patients with severe sepsis [14, 15]. Recently, we reported that rTM might improve severe ARDS complicated with DIC in pediatric patients [16]. In the present case, after rTM administration, OI gradually improved and the DIC score also decreased without bleeding-related or other adverse events. The levels of sRAGE and HMGB-1, biomarkers of ARDS, decreased after administration of rTM. sRAGE is a biomarker of Type I alveolar epithelial cell injury, and plasma levels of sRAGE increase in patients with acute lung injury [17]. HMGB1 is considered to be one of the late mediators and damage-associated molecular patterns (DAMPS) contribute to innate immunity [18]. In addition, the serum levels of INF-γ and IL-6 also decreased after the administration of rTM. We previously reported that serum level of INF-γ on the intubation day was one of the possible prognostic factors affecting a fatal outcome in a child with severe ARDS of pulmonary origin [19]. On the basis of the previous results, poor outcome might be predicted in the present case. Nevertheless, the patient recovered from severe conditions, and inflammatory responses were attenuated after administration of rTM. Although it might be difficult to determine what specific intervention could be the most effective for very severe conditions, rTM might be involved in improvement of severe ARDS via anti-inflammatory effects.

In conclusion, we have reported successful treatment of TB pneumonia-induced severe ARDS complicated with DIC using multimodal interventions including cardiopulmonary supports, anti-tuberculous therapy, and administration of immunoglobulin and rTM in a female child.

## Abbreviations

ALT: Alanine aminotransferase
ARDS: Acute respiratory distress syndrome
AST: Aspartate aminotransferase
BUN: Blood urea nitrogen
CMV: Cytomegalovirus
Cre: Creatinine
CRP: C-reactive protein
DAMPS: Damage-associated molecular patterns
DIC: Disseminated intravascular coagulation
HFO: High frequency oscillating ventilation
HIV: Human immunodeficiency virus
HMGB-1: High mobility group box 1
IVIG: Intravenous immunoglobulin
OI: Oxygenation index
PF ratio: PaO_2_/FiO_2_ ratio
PICU: Pediatric intensive care unit
PLT: Platelet
PT: Prothrombin time
RBC: Red blood cell
rPCR: Real-time polymerase chain reaction
rTM: Recombinant human soluble thrombomodulin
sRAGE: Soluble receptor for advanced glycation end products
TB: Tuberculous or tuberculosis
TLF: Tracheal lavage fluid
WBC: White blood cell
